# Characterizing Breakthrough Cancer Pain Using Ecological Momentary Assessment with a Smartphone App: Feasibility and Clinical Findings

**DOI:** 10.3390/ijerph18115991

**Published:** 2021-06-03

**Authors:** Francisco Villegas, Verónica Martínez-Borba, Carlos Suso-Ribera, Diana Castilla, Irene Zaragoza, Azucena García-Palacios, Carlos Ferrer

**Affiliations:** 1 Consorcio Hospitalario Provincial de Castellón, Pain and Radiotherapy Units, 12002 Castellón, Spain; franvilles@hotmail.com (F.V.); carlos.ferrer@hospitalprovincial.es (C.F.); 2 Department of Basic and Clinical Psychology and Psychobiology, Universitat Jaume I, 12071 Castellón, Spain; borba@uji.es (V.M.-B.); azucena@uji.es (A.G.-P.); 3 Instituto de Investigación Sanitaria de Aragón, 50009 Zaragoza, Spain; 4 Department of Personality, Assessment, and Psychological Treatments, Universidad de Valencia, 46010 Valencia, Spain; diana.castilla@uv.es; 5 CIBER of Physiopathology of Obesity and Nutrition (CIBERON), 28029 Madrid, Spain; irenezaragoza@gmail.com

**Keywords:** breakthrough cancer pain, ecological momentary assessment, smartphone application, pain management, pain characterization

## Abstract

Background: mobile applications (apps) facilitate cancer pain ecological momentary assessment (EMA) and provide more reliable data than retrospective monitoring. The aims of this study are (a) to describe the status of persons with cancer pain when assessed ecologically, (b) to analyze the utility of clinical alarms integrated into the app, and (c) to test the feasibility of implementing an app for daily oncological pain monitoring. Methods: in this feasibility study, 21 patients (mean age = 56.95 years, SD = 10.53, 81.0% men) responded to an app-based evaluation of physical status (baseline and breakthrough cancer pain (BTcP)) and mental health variables (fatigue, mood, and coping) daily during 30 days. Results: cancer pain characterization with the app was similar to data from the literature using retrospective assessments in terms of BTcP duration and perceived medication effectiveness. However, BTcP was less frequent when evaluated ecologically. Pain, fatigue, and mood were comparable in the morning and evening. Passive coping strategies were the most employed daily. Clinical alarms appear to be useful to detect and address adverse events. App implementation was feasible and acceptable. Conclusion: apps reduce recall bias and facilitate a rapid response to adverse events in oncological care. Future efforts should be addressed to integrate EMA and ecological momentary interventions to facilitate pain self-management via apps.

## 1. Introduction

Breakthrough cancer pain (BTcP) is a heterogeneous construct whose definition is far from clearly established [1]. However, it is generally accepted that BTcP includes two main components, namely (i) the presence of controlled baseline oncological pain and (ii) a transient exacerbation of this pain [2,3]. Accordingly, BTcP was recently defined as a severe pain episode of short duration (usually less than 30 min) in patients who are receiving stable opioid-based analgesics for baseline pain, which is described as mild to moderate in intensity (i.e., under four points in a numerical rating scale from 0 to 10) [4]. According to its triggering factors, BTcP could be classified as incidental (predictable: provoked by voluntary movements such as walking; unpredictable: provoked by involuntary reactions such as swallowing or intestinal distension) or spontaneous (i.e., without a clear trigger) [1,2].

The prevalence of BTcP has oscillated across studies due to the heterogeneity of assessed samples and the lack of a clear definition of BTcP [5], but it is estimated that, on average, it affects around 60% of oncological patients [6]. Due to its high prevalence rates, researchers have focused on analyzing the specific characteristics of BTcP episodes. It seems that BTcP can occur up to 3–4 times a day and reaches its intensity peak in less than 10 min (but, if untreated, it can last up to 45–60 min) [4,7]. In general terms, reports of BTcP episodes have indicated moderate to severe intensity levels, with studies reporting mean pain intensity scores around 7.5/10 [8,9].

BTcP significantly impacts the quality of life and the emotional and functional status of patients with cancer, as well as the dissatisfaction with the treatment and the costs associated with healthcare provision [10,11,12,13,14]. Not surprisingly, it was postulated that the assessment of BTcP is a key element in pain management in persons with cancer and should be incorporated in routine oncological care [4]. Thus, monitoring of patients with cancer should incorporate the evaluation of both baseline pain and BTcP episodes, including not only the presence/absence of BTcP but also the characteristics of the episodes [1,15]. This information (i.e., periodicity, duration, and intensity of BTcP episodes) could be especially relevant for clinicians who attend to people with pain complaints, as it may help guide care plans [4] and provide personalized treatments [16].

In the last decade, several studies have explored the characterization of BTcP cross-culturally [9,12,14]. While acknowledging the relevance of these studies, some limitations emerge from the existing literature. First, descriptive studies have generally relied on retrospective measures that could be influenced by memory biases and imprecision. For example, research has found that patients report more severe symptoms (i.e., higher pain intensity and fatigue) when reported retrospectively compared to real-time data [17]. Second, studies that explored the efficacy of pharmacological treatments for BTcP management are clinical trials [18] with a reduced number of cross-sectional assessments (i.e., pre-, post-treatment, and a reduced number of follow-ups). This is problematic because pain characteristics and disease status are in continuous change over time, even within days [4,19]. Therefore, neither a retrospective assessment nor a reduced number of episodic assessments of pain status might provide a reliable and valid picture of the BTcP phenomenon. As a solution to this, Ecological Momentary Assessments (EMA), which involves real-time repeated assessments and has the potential to reduce recall bias and facilitates personalized care according to patients’ responses, has emerged as the preferred methodology for BTcP management [20,21].

The need for EMA in persons with cancer, however, does not only apply to pain reports in general and BTcP in particular. The life expectancy in oncological patients has increased dramatically thanks to medical advances in the treatment of this disease. Therefore, outcomes associated with quality of life should be incorporated in the routine assessment of persons with cancer [22]. This includes, but does not limit to, baseline pain and BTcP. As such, guidelines specialized in oncological care recommend the assessment not only of pain intensity but also other health-related variables that could negatively impact oncological patients’ quality of life and pain intensity, namely fatigue, mood (anxiety and depressive symptoms), and coping strategies, among others [16,23,24,25]. Different studies have explored the role of these variables in oncological pain context and support their influence on pain and quality of life status [26,27,28]. However, as in the case of BTcP characterization, these studies were cross-sectional and retrospective, which does not allow identifying how people manage pain in their real context and, as a consequence, it is not possible to provide psychological support in real time (e.g., ecological momentary intervention) [29].

Despite the advantages of EMA, its implementations in health settings in general and cancer management in particular have been challenging and infrequent. For example, health care services still generally rely on face-to-face interventions to manage pain conditions (even though the COVID-19 pandemic has imposed some changes in this regard) [30]. In terms of implementation, it is not feasible (economically and practically) to conduct EMA via face-to-face only as it would imply the patient traveling to the hospital as well as human and material resources (i.e., clinical staff available to conduct the assessments and a physical place to conduct them). Additionally, this could not be considered a naturalistic assessment. In an effort to integrate EMA into the home, other solutions included telephone and paper-and-pencil assessments. However, phone calls still require high economic and personal involvement, and non-compliance issues with paper diaries have frequently emerged [31,32]. As an alternative to face-to-face, paper diaries, and telephone assessments, the use of electronic assessment has emerged as a potential solution to conduct EMA with reduced patient and healthcare system burden [33]. Additionally, electronic EMA results in more reliable information when compared to paper-and-pencil assessments [17].

The implementation of technological solutions for EMA, such as treatment/evaluations delivered through the internet or via smartphone applications (apps), has demonstrated to be useful for clinical practice [34,35] and has resulted in high satisfaction as reported by sanitarians [3] and end-users [36]. This is extremely important because technologies need to be useful as well as acceptable, which means that end-users and other relevant stakeholders are willing to use them. Particularly, apps seem to be very convenient tools for EMA due to their high availability in the daily lives of the majority of individuals, at least in industrialized countries. For example, it is estimated that 73% of Europeans aged between 16 and 74 years use mobile devices (i.e., smartphone, tablet, PDA, etc.) to access the internet, and 55% of them used the internet to seek health-related information [37]. By attending to these data, the use of apps might be feasible to implement EMA in BTcP management. Additionally, as indicated in past research, the use of such apps for EMA could facilitate the implementation of clinical alarms embedded in the app, which could help to detect and respond to undesirable events (e.g., side effects of the medication or treatment non-adherence), which could ultimately improve quality of life in this population [38].

Several apps already exist to facilitate the management of numerous health conditions [39,40,41], and specifically cancer pain and BTcP [3,42]. In the specific field of pain, despite the increased interest in app development, reviews have generally found that there is still poor evidence to support the use of most mobile applications [43,44]. It seems that the apps’ content is not validated, most studies do not specify whether pain experts were involved in the development of these devices, security and interface problems are frequent, and psychological assessments are generally not included. The latter is especially problematic because psychological factors are highly important in oncological pain care [16,23,24,25]. Therefore, in the present study, we decided to use Pain Monitor, a validated app for the EMA of pain and related outcomes (e.g., fatigue, mood, and coping) [45]. The app, which was recently tested in a randomized controlled trial [38], involved pain experts in its development, was tested for interface problems (patient acceptability), and follows the current regulations in terms of data safety [45].

The aims of this study are: (i) to characterize the patient status in terms of pain (i.e., baseline and BTcP) and related factors (mood, fatigue, and coping strategies) when assessed ecologically and daily during a certain time period (i.e., a month in the present study); (ii) to explore the frequency and impact of clinical alarms (i.e., responses prompted in the medical staff) when detected by the app, and (iii) to test the feasibility of implementing an app-based monitoring system for EMA in patients with cancer pain (i.e., adherence rates and end user’s and healthcare professionals’ satisfaction with the app-based EMA). We expect (i) to replicate the findings from past research [9,12,14] when characterizing patients with cancer (e.g., frequency, duration, and severity of BTcP and characteristics of baseline pain). In addition, based on recent research with persons with musculoskeletal pain [38], (ii) we expect to obtain clinical alarms with the app, and we anticipate that the medical team will be willing to respond to them (e.g., calling the patient and providing support or a change in the treatment). Finally, according to past research [45], (iii) we expect that the implementation of the app for EMA will be feasible (i.e., the number of patients that can use the app, adherence to it, and satisfaction with app-based EMA by patients and healthcare professionals).

## 2. Materials and Methods

This was a feasibility clinical trial to evaluate whether the implementation of an app to monitor patients with cancer pain daily provided novel data about the characteristics of cancer breakthrough pain and the frequency of undesired clinical events during treatment for cancer thanks to the alarms in the app. In addition, we aimed to explore whether the use of this monitoring tool was viable and resulted in actions by the medical team that could potentially improve the quality of life of the participants. The study was previously registered in Clinicaltrials.org (NTC03597737).

### 2.1. Procedures and Sample

Eligibility criteria included being 18 years or older, having the physical and mental capacity to participate in the study, having cancer pain, holding a mobile phone with an Android operating system (the app was only available for Android at the time of the study for economic reasons and because of the differences in the penetration between Android and other operating systems in Spain), and signing the informed consent form.

Potential participants were patients experiencing disease-related pain with an appointment at the cancer unit (blinded) during 2017 and 2018. Individuals who meet the requirements to participate in the study of age and cancer were invited to participate by the medical team of the cancer unit. In a first step, twenty-five participants were screened for eligibility criteria. Four of them did not meet inclusion criteria as they did not have a mobile phone with an Android operating system. Thus, the final sample comprised of 21 participants. The sample size was calculated according to previous recommendations for feasibility studies, which suggest a minimum of 12 participants per group [46].

A researcher that was hired to provide assistance with the project helped the participants install the app and make the first use. The app can be downloaded for free in the Play Store (https://play.google.com/store/apps/details?id=doloronco.code, accessed on 3 May 2021), but an individual code to access the app is needed for its use (this is provided by the physician). The participants were asked to use the app daily for 30 days, a period of time that was believed to be sufficient to obtain a picture of the pain profile of the participants and the difficulties experienced with introducing this assessment tool in the daily life of individuals with cancer.

All of the participants in the study received the usual treatment for their pain and condition. However, if an alarm was received, the medical team responsible for the treatment could decide to use the clinical alarms (see a description below) to make changes to the treatment.

### 2.2. Measures

In this study, we used the assessment protocol validated in the Pain Monitor app [45]. A few adaptations, however, were made to ensure that the content was relevant to the population of the study (persons with cancer). The assessment protocol with the adaptations was named “Pain Monitor—breakthrough pain”.

#### 2.2.1. Daily Assessment with the App

As in previous research, patients were prompted twice daily, in the morning and evening, because the experience in relation to some items can change depending on the time of the day (i.e., the severity of pain or fatigue), while the assessment of some items was more sensible in the morning (i.e., interference of pain on sleep) or in the evening only (i.e., interference of pain on daily social activities).

The ecological momentary evaluation included previously validated single items of treatment adherence, pain severity, fatigue, mood, relief of pain treatment, and use of coping strategies to deal with the pain [45]:

- Treatment adherence: Daily treatment intake was measured with a single item, “Have you taken the pain medication prescribed by your doctor today?”. Response options are: (a) “Yes, I have taken it”, (b) “No, but I will take it”, and (c) “I have not taken it and I will not take it today”.
- Pain severity: This item assesses the current level of pain (“Please indicate your current level of pain”). Responses range from 0 = “No pain” at all to 10 = “Extreme pain”.
- Fatigue: This item evaluates fatigue intensity (“Please indicate your current level of fatigue”). Responses range from 0 = “No fatigue at all” to 10 = “Extreme fatigue”.
- Mood: Three items were employed to assess the intensity of happiness (“Please indicate your current level of happiness”), sadness (“Please indicate your current level of sadness”), and anxiety (“Please indicate your current level of anxiety”). The three items use a similar response scale ranging from 0 = “No happiness/sadness/anxiety” at all to 10 = “Extreme happiness/sadness/anxiety”.
- Perceived utility of pain treatment: This item evaluates the perceived pain relief after the baseline pain medication intake (“Please indicate to what extent the baseline medication relieved your pain today”). Responses range from 0% = “No relief at all” to 100% = “Complete relief”.
- Coping strategies: we explored the coping strategies implemented by patients on a given day (“Select the coping strategies that you have used today to cope with pain. You can choose more than one option”). The following list of coping strategies was presented to the patients: inactivity, relaxing, talking to somebody, physical activity, coping self-statements, distracting/ignoring the pain, or praying. This list was created after reviewing the most frequent coping strategies employed in pain research and was validated to be used with the app in a previous investigation [45].

#### 2.2.2. On-Demand Assessment of Breakthrough Pain with the App

**Figure 1:**
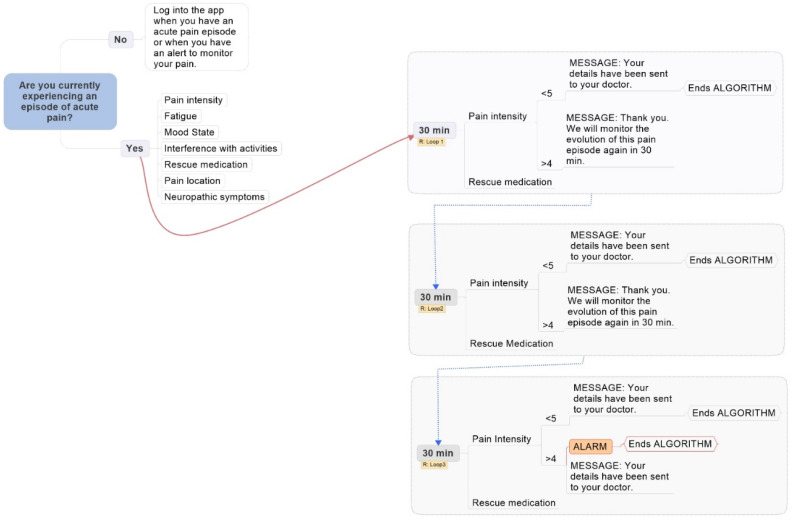
On-demand, breakthrough pain assessment algorithm.

In addition to the morning and evening assessments, which were identical to the ones in the original app Pain Monitor, the novelty of the “breakthrough pain” version of the app was the inclusion of a breakthrough pain on-demand evaluation. The algorithm used in the breakthrough pain assessment is depicted in Figure 1. When patients access the app outside the pre-established times, the app asks them whether they want to report a current breakthrough pain episode. If the answer is no, they are asked to access the app only for planned evaluations or reporting breakthrough pain episodes. If the answer to the question, however, is positive, a first assessment is launched to evaluate the characteristics of the current episode in terms of pain severity and interference in functioning, fatigue, mood, current or planned use of rescue medication, pain location, and neuropathic characteristics of the pain.

A maximum of three loops might then occur 30 min after the same assessment and then every 30 min if the pain severity levels are still equal or greater than 5. At the beginning of each loop, the patient is asked about the severity of the current pain to check whether the breakthrough pain episode ended. If the reported pain severity is 4 or less, the loop closes. On the contrary, if the pain severity is still 5 or over on a 0–10 scale, the patients are asked to report the amount of rescue medication used or whether they plan to take medication if the pain persists. They are then told that the app will inquire them again about their pain status 30 min later. In total, three loops can be potentially shown to the patient, each questioning about pain severity and rescue medication use. The loops only close if patients report an amelioration of the pain levels (4 or less). If the pain of 5 or over persists after the third loop, the assessment ends and a clinical alarm is sent to the medical team.

#### 2.2.3. App Usability and Satisfaction (Evaluated by Phone or by Paper-and-Pencil)

App-related outcomes were either evaluated by phone (patients) or by paper-and-pencil (clinicians). To assess the usability of the app, the 10-item System Usability Scale (SUS) [47] was administered approximately ten days after the first day of app use to the patients (it is recommended to evaluate acceptability and usability relatively soon to detect problems early and to avoid underestimation of problems due to repeated use). A member of the team called the patients by phone. The SUS is a widely-used questionnaire that assesses the perception that a tool is simple and useful (e.g., “I needed to learn many things before I could start using the system” or “I found the system easy to use”). Response scales have a 1 (Completely disagree) to 5 (Completely agree) range, and half of the items are recoded so that higher scores in the scales represent greater usability and acceptability. The total score is multiplied by 2.5 so that scores range from 0 = Worst imaginable” to 100 = “Best imaginable”. In previous research with the original version of the app, which uses the same graphical interface as the one used in the present study, found that 100% of the persons with chronic pain (*n* = 40) found the app easy to use, and 92.7% of them found it useful [45].

To evaluate the satisfaction of the professionals, an assessment protocol used in previous similar research [45] was administered onsite at the end of the study to the medical team involved in the study at the cancer clinic (*n* = 2). The responses for questions addressing satisfaction with the app in the medical team (i.e., “I think that the use of alarms improves the safety of breakthrough cancer pain treatments” ranged from 1 = “Completely disagree” to 5 = “Completely agree”. Higher scores indicate greater satisfaction with the app.

#### 2.2.4. Clinical Alarms

In order to explore whether the safety of the patients could potentially be enhanced with ecological momentary assessment via app, we set a number of clinical alarms together with the medical team of the cancer unit. An email was sent to the medical team whenever an alarm was detected by the app. The patient was blinded to the clinical alarms. Therefore, the patients were not notified about the alarm when this was generated in the app. This was to avoid creating the expectation that an action by the medical team would occur. In addition, this also minimizes the risk of using the app responses for personal interests (i.e., to obtain lower/higher medication doses, to increase the number of face-to-face appointments, etc.). Whenever the medical team considered that an action was necessary for an alarm, they responded to it by calling the patient the following working day after the alarm was generated. The clinical alarms were similar to those used in previous research [38] but were reduced to a more manageable set according to the anticipated problems and needs of the target population:

- Baseline pain > 5 for two consecutive days;
- Vomiting/nausea for two consecutive days;
- More than four episodes of breakthrough pain in 1 day;
- Breakthrough pain lasts for over 90 min.

### 2.3. Data Protection Policies in the App

The app has several layers of access security to prevent unwanted access to the system. These systems are based on the encryption of the channel through certificates and the randomization of an access key each time the connection is made.

The database provided by the app is completely anonymous since the system does not store any personal information. However, the application collects the International Mobile Equipment Identity (IMEI), which is a unique reference for each mobile phone. This number was used to link the responses in the database to a participant. To guarantee confidentiality, the storage of the IMEI of the participants was external to the system and was stored locally in the computer of the cancer clinic. Only the medical team could relate the IMEIs of the patients with their personal data (name and surname, address, telephone number).

All the data collected by the app comply with the guidelines established in the laws and data protection regulations in force.

### 2.4. Data Analysis

The analysis of the data included a description of the means and standard deviations of pain severity, fatigue, and mood outcomes throughout the study, as well as the frequency and characteristics of breakthrough pain episodes. First, sociodemographic and pain characteristics (i.e., pain location, duration, and treatment) were analyzed to describe the sample. Second, mean and standard deviations were calculated for daily measures of pain, fatigue, and mood (i.e., happiness, sadness, and anxiety). Because these variables were asked twice a day, a Student *t*-test was conducted to compare morning-to-evening pain, fatigue, and mood scores. Then, BTcP episodes were characterized in terms of frequency (i.e., number of episodes reported), time of onset, and average pain intensity.

Regarding the perceived utility of baseline medication, we calculated the proportion of time that patients reported high pain relief (70–100% of perceived medication utility in pain reduction), low pain relief (between 0 and 30% of perceived medication utility), and moderate pain relief (between 40 and 60% of perceived medication utility). The same descriptive analyses were conducted to explore the frequency of use of strategies to cope with pain.

The information from the clinical alarms is reported. We indicate the type of clinical alarm, as well as the response provided by the professionals. Finally, feasibility and satisfaction outcomes are explored. In particular, we report the participants’ adherence with the app (number of missing reports) and the app satisfaction results (based on the SUS questionnaire described previously). The anonymous database will be made available under reasonable request.

## 3. Results

### 3.1. Sample Characteristics

Twenty-one patients met the inclusion criteria and were included in the study. Of these, 17 were men (81.0%) and 4 were women (19.0%). Their mean age was 56.95 years (SD = 10.53; range = 25–85). The vast majority of participants were married or in a relationship (*n* = 16, 76.2%). Only one participant was working at the time of study recruitment. The remaining participants were either on sick leave (*n* = 5, 23.4%), permanent disability (*n* = 7, 33.3%), or retired (*n* = 8, 38.1%). Regarding educational level, the majority of participants had elementary or no studies (*n* = 13, 61.9%), four participants had completed secondary education, and four participants had tertiary education.

Only three patients reported having a formal diagnosis of major depressive disorder (*n* = 2, 9.5%) or anxiety disorder (*n* = 1, 4.8%).

### 3.2. Cancer Pain Status and Treatment at Study Onset

The participants reported that their major focus of cancer-related pain occurred in the shoulders (*n* = 9), head (*n* = 4), neck (*n* = 4), low back (*n* = 2), abdomen (*n* = 1), and thorax (*n* = 1). Cancer pain duration was generally short (i.e., less than 6 months; *n* = 9), but a significant number of patients had been experiencing pain for longer periods (i.e., between 1 and 5 years; *n* = 7). The remaining patients reported experiencing pain for between 7 and 12 months (*n* = 3), or over 5 years (*n* = 2).

In terms of treatment for their pain, patients were asked to differentiate between the treatments they received for their baseline pain and the treatment they had been prescribed for their breakthrough pain episodes (rescue medication).

Regarding baseline pain, 20 participants were receiving pharmacotherapy (95.2%), 3 were receiving infiltrations (14.3%), 2 were receiving physiotherapy (9.5%), and 2 were receiving psychotherapy (9.5%). None of the participants were taking natural/alternative treatment for their baseline pain. Pharmacotherapy for their baseline included morphine (*n* = 11, 52.4%), fentanyl (*n* = 6, 28.6%), tramadol (*n* = 3, 14.3%), or oxycodone (*n* = 1, 4.8%). Routes of medication administration were either oral (*n* = 12), transdermal (*n* = 15), or intravenous (*n* = 1). Regarding adherence with baseline medication, 88.70% of the times that patients responded to the adherence question, they reported that they had already taken the medication. The remaining 11.30% of times, the patients responded that they had not taken it yet, but they would take it later. None of the participants ever indicated that they were not willing to take the baseline medication that day.

Regarding the breakthrough pain, all patients reported having been prescribed pharmacotherapy. This included fentanyl (*n* = 18, 66.7%) and morphine (*n* = 3, 14.3%). The routes of medication administration were all oral (*n* = 21).

In terms of treatment for cancer, six participants were receiving chemotherapy (28.6%) and 11 were receiving radiotherapy (52.4%).

### 3.3. Evolution of Patient Status during the Study as Reported Daily in the App

**Figure 2:**
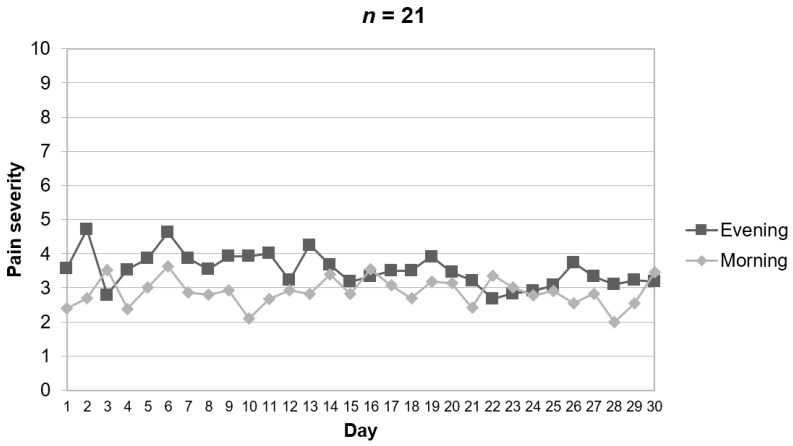
Evolution of baseline pain in the morning and evening during the study based on ecological momentary assessment with the app.

**Figure 3:**
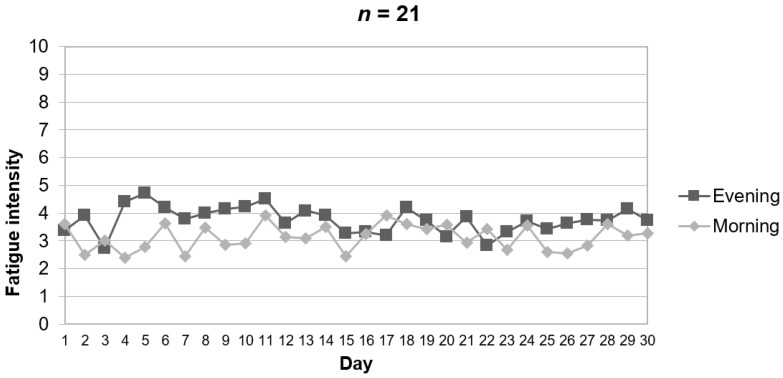
Evolution of fatigue in the morning and evening during the study based on ecological momentary assessment with the app.

The clinical characteristics of the sample are reported in Table 1. The daily assessment of baseline pain revealed an average morning and evening pain of 2.9 (SD = 2.1) and 3.5 (SD = 2.3), respectively. The graphical representation of baseline daily pain revealed that pain was often more severe in the evening than in the morning (Figure 2). This difference was not significant and small in size (*t* [40] = 0.88, *p* = 0.383, *d* = 0.27). Similar to pain severity, the average fatigue reported by the participants was higher in the evening (mean = 3.8, SD = 2.4) than in the morning (mean = 3.1, SD = 2.4). Again, this difference was non-significant and small in size (*t* [40] = 0.95, *p* = 0.350, *d* = 0.29). Figure 3 shows a representation of daily average scores in fatigue in the sample. Table 1 also shows the sample characteristics in relation to mood states (i.e., happiness, sadness, and anxiety). A graphical representation of daily happiness, sadness, and anxiety is provided in Appendix A, Appendix B and Appendix C.

### 3.4. Breakthrough Pain Episodes and Characteristics

The characteristics of breakthrough pain episodes are reported in Table 2. During the study (30 days), nine patients reported having at least one breakthrough pain episode (42.86%). Of these, the majority of patients reported experiencing one episode only (*n* = 6), while the remaining three patients indicated six, four, and three episodes, respectively. The duration of episodes was generally shorter than 30 min (*n* = 14). The remaining episodes had a duration of between 30 and 60 min (*n* = 4) and between 60 and 90 min (*n* = 1).

The average severity of pain during breakthrough episodes was higher at the initial stage of the episode, that is, at onset ($\;\bar{x}\;$ = 6.95, SD = 0.71) and decreased with time. The mean pain scores 30 and 60 min after episode onset (loops 1 and 2) were 4.11 (SD = 1.97) and 3.40 (SD = 1.14), respectively. Only one patient reported sufficiently high pain (>4) at loop 2 to receive the third loop of questions.

### 3.5. Perceived Utility of the Baseline Medication for Pain during the Study

**Figure 4:**
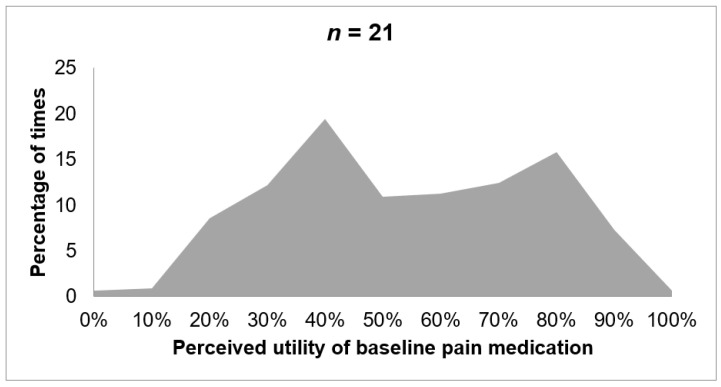
Perceived relief of baseline medication for pain.

The daily assessment of the perceived utility of the baseline pain medication is presented in Figure 4. The *x*-axis represents the perceived utility of baseline medication for pain relief. The *y*-axis indicates the percentage of time that a given utility is selected. As observed in Figure 4, 36.17% of the times that patients responded to the perceived utility of baseline pain medication item, they indicated a high relief in pain intensity (between 70% and 100% of medication utility). The lowest pain relief (between 0% and 30% of perceived medication utility) was reported 22.19% of times, while a moderate relief of pain intensity (between 40% and 60% of perceived medication utility) was indicated 41.64% of the times patients were asked in the app.

### 3.6. Use of Daily Strategies to Cope with the Pain

**Figure 5:**
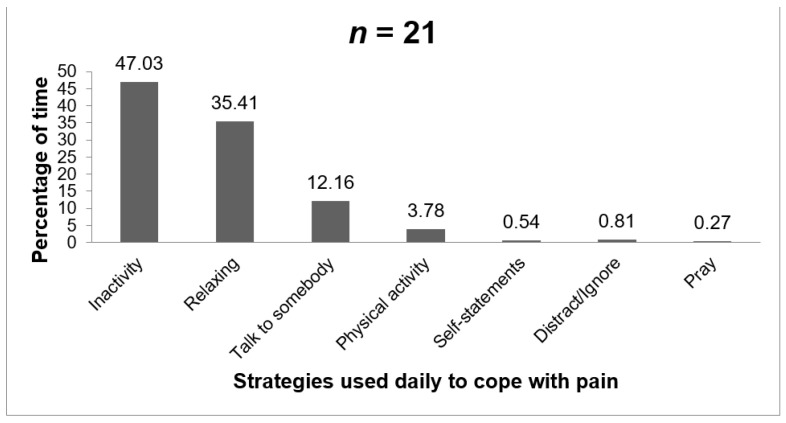
Use of strategies to cope with the pain (aggregate of daily reports).

The distribution of coping strategies used daily is graphically represented in Figure 5. The coping strategies are presented on the *x*-axis. The *y*-axis indicates the proportion of times that a given strategy is selected across the 30-day study. In the presence of pain, patients preferred to remain inactive (47.03% of times) or to relax (35.4% of times). The remaining strategies included talking to somebody (12.16%), performing physical activity/stretching (3.78%), distracting/ignoring the pain (0.81%), using coping self-statements (0.54%), and praying (0.27%).

### 3.7. Alarms

The date of the alarm, the patient ID, the type of alarm, and response to it by the healthcare team are reported in Table 3. In total, 27 alarms were received from 9 patients. Therefore, alarms were not received by approximately half of the sample (*n* = 12). Alarms referred to excessive baseline pain severity (*n* = 8), sleepiness (*n* = 17), vomiting (*n* = 1), and nausea (*n* = 1). Responses to the alarms ranged from telephone contact to provide medical advice (*n* = 13), arrange an appointment (*n* = 5) to increasing the medication (*n* = 2), or hospitalization (*n* = 1). On four occasions, the attempts to contact the patient were unsuccessful.

Breakthrough pain episodes were reported at all times of the day, including morning (*n* = 5), afternoon (*n* = 7), and evening (*n* = 7).

### 3.8. Feasibility

In terms of adherence, the majority of participants (*n* = 13, 61.9%) used the app daily during the 30 days of study. The remaining participants used the app during 25 (*n* = 1), 24 (*n* = 1), 18 (*n* = 1), 17 (*n* = 1), 14 (*n* = 2), and 8 days (*n* = 2).

Four participants did not have an Android operating system and could not be included in the study. There were no technical problems once it was installed, as reported during the control call that occurred ten days after the installation of the app (this was when the SUS was administered).

### 3.9. App Usability and Satisfaction (Patients and Medical Staff)

The mean score of the System Usability Scale in patients was 85.77 (SD = 12.09). The average score of this study falls within the 4th quartile, indicating very good acceptability by users and placing our application between excellent and best imaginable in the adjective rating scale [48].

The two clinicians of the cancer unit that participated in the study completely agreed that the app improves the safety of treatments for BTcP, that the alarms of app improved the effectiveness of treatments for BTcP, that the alarms of app improved the adherence to treatments for BTcP, that the app would be useful for them as healthcare professionals, that the app was useful for patients with BTcP, that the app could be integrated into routine care, and both would like to continue using the app. One of them mostly agreed that he felt safer with the app when treating BTcP, while the other was more enthusiastic and completely agreed. One indicated that the use of alarms resulted in no burden for him, while the other indicated a certain degree of burden. They indicated that the additional daily time they required to manage app-related clinical alarms ranged from 20 to 30 min.

## 4. Discussion

The main objective of the present study was to use a mobile application to provide an accurate characterization of the patients’ physical and psychological status. We also aimed to explore the utility of clinical alarms, as well as to test the feasibility of using an app for EMA in cancer pain management. Overall, our results indicated that EMA provided similar pain characterizations compared to previous studies that used retrospective paper-and-pencil assessments. We also obtained a daily characterization of psychological variables and a measure of fatigue, which is relatively novel to the literature in cancer pain. In addition, we found clinical alarms to be useful for the evaluation and management of treatment side effects. Finally, the results support the idea that the implementation of the app was feasible (i.e., high usability and satisfaction) in our sample. A summary of the key points discussed in the following lines can be found in Table 4.

Consistent with the previous literature [4], our EMA finding showed that BTcP episodes occurred in patients with moderate (<4 points) and controlled baseline pain, and it usually lasted less than 30 min. Contrary to past research, our sample reported less severe symptoms (i.e., lower severity, incidence, and frequency of BTcP). Previous studies indicated 60% of patients having BTcP 3–4 times a day with an intensity of 7.5 on a 0–10 scale [4,6,7,8,9]. Different from this, only 43% of our participants experienced BTcP episodes, which usually happened once a day and were relatively milder in severity (7 on a 0–10 scale) at the onset. The BTcP intensity was higher at onset and decreased with time. Thus, it seems that the medication for BTcP provided pain relief within the expected frame time (i.e., 15–20 min) [49,50]. Several reasons may explain these results. First, as suggested in previous research [17], it is possible that EMA reduced recall biases compared to retrospective assessments, resulting in less severe symptoms. In fact, the aforementioned study indicated that retrospective assessments generally overestimated daily pain severity status, which would be consistent with our findings. However, note that our study does not compare the pain characterization of patients assessed retrospectively vs. patients evaluated with an app. Therefore, this conclusion is simply speculative at this stage and should be tested in a larger study comparing these two assessment methods in two separate groups. An alternative explanation, however, lies in the use of technology and reliance on individual motivation to report BTcP in real-time. In this sense, the fact that half of the patients did not report any clinical alarm could be interpreted as indicating that they did not experience important unwanted clinical events. However, it is also possible that the patients decided not to register the negative events they were experiencing (e.g., BTcP episodes) in the app. The latter was not explored in detail, so the actual reason for the findings remains unclear. Finally, the high subjective perceived utility of baseline medication (i.e., during 78% of the time, patients reported 40–70% of pain relief) could indicate that pharmacological treatment (e.g., morphine, fentanyl, tramadol, and oxycodone) was adequate in our sample and it maintained pain intensity in acceptable levels for the patients. This is important because it was found that between 13% and 23% of patients are not receiving appropriate opioid therapy [51]. Fortunately, characterization of both baseline and BTcP episodes may help to detect when pharmacotherapy is not working well or when pain relief is not good enough for patients. Ultimately, this may help adjust pain treatment if necessary.

In addition to BTcP, a contribution of the study was that fatigue was assessed daily during the whole study period (30 days). Fatigue is one of the most prevalent and distressing effects of cancer treatment and is defined as a subjective sense of physical, emotional, and cognitive exhaustion related to cancer disease, which negatively interferes with the patient’s functioning and quality of life [23]. Similar to pain intensity, our results showed that fatigue was comparable in the morning and evening, even though a visual inspection indicated slightly higher levels of fatigue in the evening. There is literature on the end-of-dose effect in pain medications [52] (i.e., pain increases when a scheduled dose finishes). This, together with increased tiredness caused by daily living activities (i.e., household tasks, childcare, job activities, etc.) [53], might negatively impact the patients’ perceived fatigue at the end of the day. This was not the case in our study, even though the visual analysis suggested more fatigue in the evening. In this sense, a more specific analysis of individual data could help us to determine the specific patient’s needs and to provide personalized multimodal treatments, which include education on cancer-related fatigue, prescription on exercise, and cognitive therapy [24,54]. Because fatigue seems to be related to pain intensity [55], different psychological components could be used to address both pain and fatigue. For instance, psychoeducation could help to learn about inadequate positions and efforts during the day, energy preservation, task prioritization, and how to delegate non-important tasks [24]. Furthermore, cognitive-behavioral interventions could help to discuss dysfunctional thoughts about pain, fatigue, and activity patterns [23].

In addition to the physical characterization of cancer pain, we evaluated psychological variables that were argued to be relevant in persons with cancer pain [16,23,24,25]. Again, morning-to-evening comparisons did not reveal significant differences in mood levels. However, and similar to pain severity and fatigue, a visual inspection of the data supported the idea that slightly higher sadness and anxiety and lower happiness were reported in the evening. Studies with larger samples are needed to provide more robust findings in this regard. The literature supports an association between psychological factors and pain intensity and fatigue [26,27,28,55]. Therefore, it is possible that an increase in pain in the evening negatively impacts fatigue and mood, which in turn leads to more pain in a kind of vicious cycle [56]. Whatever the case, no causal relationships can be established from our findings due to the observational nature of the present study. Thus, even if the morning-to-evening differences were larger and significant, it would not be possible to determine whether pain intensity and fatigue indeed led to a decrease in mood status or if the opposite direction of association (i.e., mood affecting pain intensity and fatigue) is more suitable. However, it seems clear that persons with cancer may face psychological suffering, such as anxiety and depressive symptoms. This is alarming considering the low proportion of participants (*n* = 2; 9.5%) that received psychotherapy in our study. This is also inconsistent with recent recommendations suggesting that psychological assessments should include the patient’s desire for psychological support and the moral requirements that, when mental problems are detected, treatment should be offered [22,25].

Another strong point of the present study was the daily assessment of coping strategies. Several international guidelines recommend the evaluation of coping strategies as a key point of cancer pain management [16,24]. Thus, it is essential to know not only the physical and psychological symptoms that patients face (i.e., pain intensity, fatigue, anxiety, and depressive symptoms) but also to identify how they cope with these challenges. Our findings showed that passive coping strategies, such as inactivity or relaxing, were the most frequently used during the 30 days of study. In contrast, very few participants implemented more active strategies, such as conducting a physical activity or using coping self-statements to cope with the pain. These findings are consistent with previous research indicating that individuals prefer the use of passive treatment and coping strategies even though they generally believe that exercise would also help them to cope with the pain [57]. This is worrisome as passive coping strategies (e.g., reliance on others for pain control, avoiding activities due to pain, praying, or catastrophizing) are related to increased disability, anxiety, and depressive symptoms, while active coping strategies (i.e., continue to function despite the pain, engage in activities, diverting attention or exercising) could help to reduce fatigue and depressive symptoms [23,58,59]. While acknowledging this generally accepted idea that passive coping would be maladaptive and active coping would be adaptive, there is also evidence to suggest that certain strategies, such as resting or guarding, could be both adaptive or maladaptive depending on the situation (e.g., whether they are implemented to be able to gain energy and cope later or to avoid in general) [60]. In fact, studies have proposed that the problem in coping lies in the inflexibility of the selection of strategies, which would be evidenced in the present study as the repeated implementation of one or two (passive) coping strategies only [61,62]. The ability to change the coping strategy employed attending to one’s emotions has been a focus of recent interest in research. In this sense, the study by Sheppes et al. [63] found that healthy people selected different emotion regulation strategies (i.e., reappraisal or distraction) as a function of their emotion’s intensity. Particularly, people preferred to regulate emotions by using cognitive reappraisal when they faced low-intensity unpleasant emotions. On the contrary, distraction was preferred in high-intensity negative situations [63]. Therefore, based on the high frequency of use of passive coping strategies in our sample, we encourage professionals who are in continuous contact with cancer patients to provide the patients with a more varied and adaptive repertoire of coping strategies to cope with their pain (e.g., staying active, using coping self-statements, doing exercise, or resting with the aim of participating in an activity once the BTcP decreases). All this helps them manage intense, unpleasant emotions and function despite the pain, as recommended in the literature [55,57]. The clinical professionals would provide the appropriate coping strategy for mental health for these patients.

Our second objective was to assess the utility of clinical alarms. Consistent with our hypothesis, an important finding in our study was that clinical alarms, which were set in the app, are useful to detect undesirable events in the real context in which they occur. In our study, 27 alarms were detected, and 84% of them could be adequately addressed by doctors with telephone contacts, appointment re-arrangements, or medication adjustments, among others. This is especially relevant for pain management because, in the most proximal context in which patients receive care (i.e., pain and oncological units), daily assessments are not feasible. As noted in past research, when undesired effects occur (i.e., poor efficacy or side effects of the treatments), patients should not be left in charge to decide how to react in the presence of such events [64]. Note that patients, in the presence of undesired events, have numerous possible options to deal with the situation. For example, they can decide to go to the emergency services or to the general practitioner, they can try calling to the Pain Clinic, or they can decide to wait until the next appointment at the Pain Clinic. This is problematic because all of these responses could result in inadequate pain management and, additionally, is a threat to patient safety [64]. As previously suggested, the use of clinical alarms has the potential to increase the safety of patients as they allow early detection of adverse outcomes, and clinical professionals have the option to respond to them early [38]. By doing this, patients are no longer responsible for deciding whether some symptoms are acceptable or not, as this decision will rely on the clinicians and their professional judgment in front of alarms.

In relation to the latter objective of this study, that is, to test the results of implementing an app-based EMA, our findings suggest that this system was feasible. According to different implementation outcomes, we found high coverage/penetration, fidelity (adherence rates), usability, and acceptability. First, only four participants could not be engaged in the study due to the non-compatibility of their smartphone operative system. This indicates a high coverage rate because 84% of the participants that could be eligible actually participated in the study. Because the app offered to the participants was only available for the Android operative system, this coverage data were expected, and it is consistent with recent data showing that 20% of the Spanish population use iOS systems [65]. Second, high adherence rates were found. Specifically, 62% of the participants used the app during all the study period (30 days), while 81% used the app at least half of the days (≥17 days) that were indicated. This is consistent with previous findings using mobile applications for chronic and cancer pain management [38,66]. Recently, it was found that approximately 80% of participants respond to daily cancer symptom assessments, with adherence rates decreasing as the period of time using the app increased [67]. In light of the high adherence rates in the present study, it seems that patients need and appreciate being in constant contact with the clinicians. However, it is important to note that the patients in our study were contacted by clinicians only if adverse events were detected. Thus, the participants who were not called might have received less feedback about the app usefulness, and therefore their motivation to engage in the program could be reduced. Future efforts should be conducted to promote engagement with mobile apps irrespective of such calls from the clinicians. For example, they were providing positive feedback about health status to patients who did not report adverse events or including gamification when the number of daily responses augmented (i.e., health-related tips to cope with pain or unpleasant emotion management).

Similar to previous studies [68], our sample reported high scores in the System Usability Scale. This indicated that, overall, participants found the system easy to use and thought that they could use it without external help. They also believed that the app was coherent and consistent, and they felt secure using it. Technical issues were detected during phone contacts no longer than 10 days after the first day of app use. While some concerns about the appropriateness of these technologies for elderly patients were raised [69], our findings are in line with those that found that an app could be used by cancer patients regardless of their age, educational level, or user’s previous experience with technologies [68]. Finally, the clinician’s acceptability was also positive, which is an important finding for technology implementation purposes. Similar to previous studies [8,38,70], the professionals agreed that the use of the app improved treatment safety and effectiveness, and they reported that they would like to continue using the app. The analysis of both the end user’s and the clinician’s satisfaction are crucial because, if we want the patients to use mobile applications for pain management, we first need the professionals to recommend their use. In the light of our results, the implementation of this system is feasible and would provide a reliable instrument for BTcP characterization with low personal and material costs.

### Limitations

This paper provides several contributions regarding BTcP characterization and management. However, the findings should also be interpreted in the context of some limitations. First, no comparison group was included in the study. Therefore, it is not possible to establish robust differences in the patients’ characterization based on assessment tools (i.e., momentary, app-based vs. retrospective, paper-and-pencil assessments). Second, subjective information was collected with the app. This means that patients may have underestimated their symptoms or might not have been motivated to reported and, in consequence, the characterization would have been incomplete. For instance, it is possible that patients experienced BTcP every day but decided not to report the episodes because they are used to them or because they were not motivated to do it. Additionally, at the end of the study, participants were not asked about the presence of non-reported BTcP episodes, so we are not able to analyze whether non-detected BTcP episodes or alarms actually existed. Similarly, changes in the pharmacological treatment were not registered in the app, so it is not possible to explore whether changes in the medication resulted in better pain management (i.e., reduction pain intensity or decrease in BTcP frequency). Regarding alarms, despite that some of them were detected and addressed during the study period, 14% of them could not be solved because the participants could not be contacted by phone. To address this problem, we recommend that, in addition to sending the alarm to the physician, the app-based EMA should be followed by Ecological Momentary Intervention (EMI) delivered throughout the app when alarms occur. For example, a recent study found that physical exercise could be recommended as a function of the patients’ daily reports [71]. Therefore, future efforts should be conducted to provide personalized, real-time treatments that include physical and psychological strategies to favor pain self-management using different learning styles (i.e., written, audio, and videos for the self-management of symptoms) [68].

## 5. Conclusions

During the last decades, routine, real-time assessments have emerged as the gold standard for oncological pain management. Different international guidelines recommend including information beyond baseline pain and BTcP. This means assessing additional health-related variables, such as fatigue, mood, and coping strategies. Ecological momentary assessments, however, are rarely implemented in cancer management health settings. According to our results, the infrequent use of app-based assessments in our immediate context is not justified. Our results showed that the use of mobile applications is well accepted both by patients and clinicians. In addition, apps are promising solutions to overcome the barriers found in paper-and-pencil or telephone traditional assessments and allow to obtain valuable real-time information that can be used to adjust oncological pharmacological, and psychological management according to the patients’ daily responses in the app. Additionally, the inclusion of clinical alarms in mobile apps could help to increase treatment safety by detecting and rapidly responding to the adverse events that may occur with the treatment, which is crucial in oncological pain management. Finally, in terms of implementation, our results support the idea that the use of app-based systems is likely to be feasible in pain and oncological units and could be especially useful in clinical practice to guarantee the safety of patients undergoing oncological treatments.

## Figures and Tables

**Table 1:** Clinical characteristics of the sample (average scores throughout the study).

	Morning		Evening		Comparison and Effect Size		
Variables	Mean (SD)	Median	Mean (SD)	Median	*t*	*p*	*d*
Pain severity	2.88 (2.08)	2.83	3.52 (2.29)	3.55	0.88	0.383	0.27
Fatigue	3.13 (2.37)	3.03	3.75 (2.36)	3.83	0.95	0.350	0.29
Happiness	3.83 (2.33)	4.10	3.58 (2.38)	3.88	0.34	0.733	0.11
Sadness	2.38 (2.55)	1.62	2.51 (2.55)	1.97	0.17	0.870	0.05
Anxiety	1.67 (2.14)	0.73	1.65 (2.01)	0.92	0.03	0.975	0.01

SD = Standard Deviation; *t* = Student *t*-test; *p* = significance; *d* = Cohen’s *d.*

**Table 2:** Description of breakthrough pain episodes.

Patient ID	BTcP Episodes	Onset Time	Pain at Onset	Time of Loop 1	Pain at Loop 1	Time of Loop 2	Pain at Loop 2	Time of Loop 3	Pain at Loop 3	Duration of Episode
Onco0022	4	21:32	7	22:05	4					<30′
		09:27	7	10:01	4					<30′
		21:41	7	22:13	3					<30′
		08:32	7	09:04	6	09:44	4			30–60′
Onco0026	1	20:12	6	20:45	3					<30′
Onco0028	1	22:56	7	23:31	2					<30′
Onco0030	1	20:00	7	20:34	3					<30′
Onco0034	6	12:54	8	13:37	6	14:08:47	2			30–60′
		14:25	6	14:59	3					<30′
		14:19	6	15:01	6	15:32:49	5	16:04:13	3	60–90′
		13:13	7	13:49	3					<30′
		13:57	7	14:39	3					<30′
		12:56	8	13:29	3					<30′
Onco0062	3	01:53	8	2:26	9	2:58	3			30–60′
		10:39	8	11:11	3					<30′
		10:26	7	11:09	8	11:40	3			30–60′
Onco0068	1	19:04	6	19:36	3					<30′
Onco0076	1	23:57	6	00:29	4					<30′
Onco0087	1	14:35	7	15:10	2					<30′

BTcP = Breakthrough cancer pain.

**Table 3:** Description of alarms detected by the app and the response to them by the medical team.

Patient ID	Alarm	Medical Response to the Alarm
Onco0022	Baseline pain > 5 during 2 consecutive days	Telephone contact to provide medical advice
Onco0022	Baseline pain > 5 during 2 consecutive days	Telephone contact to arrange an appointment
Onco0022	Baseline pain > 5 during 2 consecutive days	Telephone contact to arrange an appointment
Onco0022	Baseline pain > 5 during 2 consecutive days	Telephone contact to arrange an appointment
Onco0022	Baseline pain > 5 during 2 consecutive days	Telephone contact to increase medication
Onco0028	Baseline pain > 5 during 2 consecutive days Vomiting during 2 consecutive days Nausea during 2 consecutive days	Telephone contact to increase medication
Onco0030	Baseline pain > 5 during 2 consecutive days	Hospitalization due to difficult pain control
Onco0034	Sleepiness/sedation during 2 consecutive days	Telephone contact to provide medical advice
Onco0034	Sleepiness/sedation during 2 consecutive days	Telephone contact to provide medical advice
Onco0034	Sleepiness/sedation during 2 consecutive days	Telephone contact to provide medical advice
Onco0034	Sleepiness/sedation during 2 consecutive days	Telephone contact to provide medical advice
Onco0061	Sleepiness/sedation during 2 consecutive days	Telephone contact to increase medication
Onco0061	Sleepiness/sedation during 2 consecutive days	Telephone contact to provide medical advice
Onco0067	Sleepiness/sedation during 2 consecutive days	Telephone contact to provide medical advice
Onco0064	Sleepiness/sedation during 2 consecutive days	Telephone contact to provide medical advice
Onco0067	Sleepiness/sedation during 2 consecutive days	Telephone contact to arrange an appointment
Onco0067	Sleepiness/sedation during 2 consecutive days	Telephone contact to increase medication
Onco0064	Sleepiness/sedation during 2 consecutive days	Telephone contact to provide medical advice
Onco0064	Sleepiness/sedation during 2 consecutive days	Telephone contact to provide medical advice
Onco0072	Sleepiness/sedation during 2 consecutive days	Unsuccessful attempts to contact the patient
Onco0072	Sleepiness/sedation during 2 consecutive days	Unsuccessful attempts to contact the patient
Onco0072	Sleepiness/sedation during 2 consecutive days	Unsuccessful attempts to contact the patient
Onco0072	Sleepiness/sedation during 2 consecutive days	Unsuccessful attempts to contact the patient
Onco0072	Sleepiness/sedation during 2 consecutive days	Telephone contact to provide medical advice
Onco0073	Baseline pain > 5 during 2 consecutive days	Telephone contact to provide medical advice

Patient ID = Patient identification code.

**Table 4:** Summary of the discussion arguments.

Main Conclusions Derived from Our Study
Ecological momentary characterization of baseline and BTcP episodes reported similar results compared to previous similar studies (i.e., BTcP pain intensity and duration)
Health-related variables, such as fatigue, mood, and coping, should be assessed in oncological care
Clinical alarms in the app were useful to detect undesirable events in the real context in which they occur. They help the professionals to rapidly detect and react to such events if necessary
App-based EMA is considered to be feasible by patients and clinicians, which supports that it can potentially be implemented in public health settings

**Figure A1:**
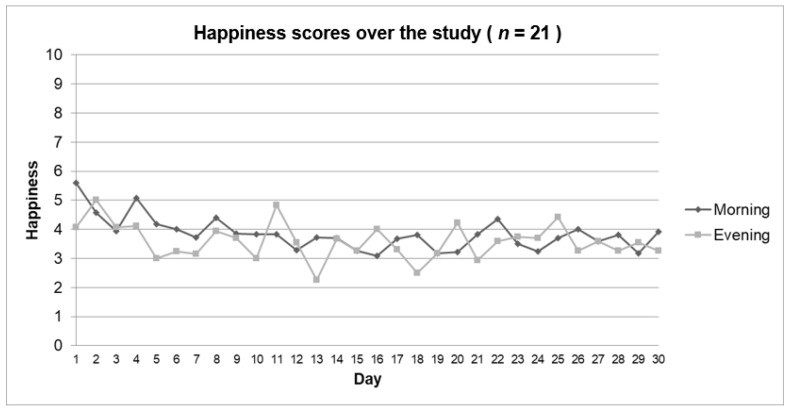
Graphical Representation of Happiness Over the Study.

**Figure A2:**
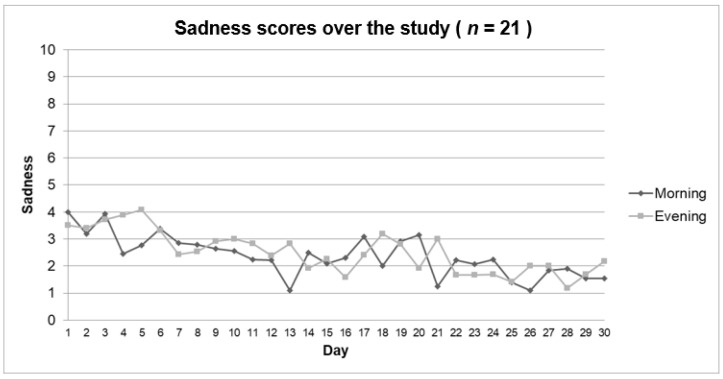
Graphical Representation of Sadness Over the Study.

**Figure A3:**
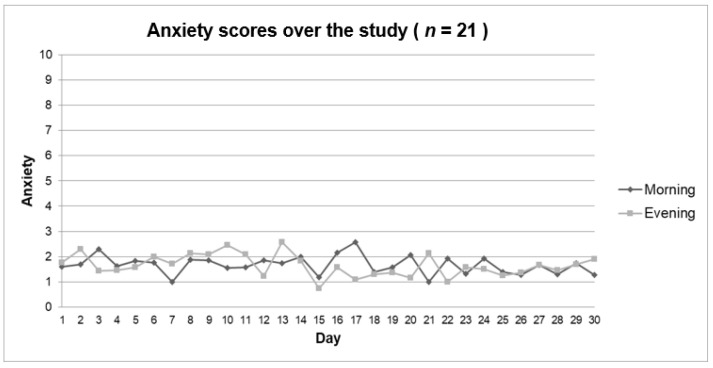
Graphical Representation of Anxiety Over the Study.

## Data Availability

The anonymous database will be made available under reasonable request.
